# Polyunsaturated Fatty Acid EAB-277^®^ Supplementation Improved Heart Rate Variability and Clinical Signs in Tracheal Collapse Dogs

**DOI:** 10.3389/fvets.2022.880952

**Published:** 2022-07-14

**Authors:** Raktham Mektrirat, Thareerat Rueangsri, Waraporn Keeratichandacha, Sasiwimon Soonsawat, Chavalit Boonyapakorn, Wanpitak Pongkan

**Affiliations:** ^1^Department of Veterinary Biosciences and Veterinary Public Health, Faculty of Veterinary Medicine, Chiang Mai University, Chiang Mai, Thailand; ^2^Integrative Research Center for Veterinary Circulatory Sciences, Faculty of Veterinary Medicine, Chiang Mai University, Chiang Mai, Thailand; ^3^Veterinary Diagnostic Laboratory, Faculty of Veterinary Medicine, Chiang Mai University, Chiang Mai, Thailand; ^4^Department of Companion Animal and Wildlife Clinic, Faculty of Veterinary Medicine, Chiang Mai University, Chiang Mai, Thailand; ^5^Veterinary Cardiopulmonary Clinic, Small Animal Hospital, Faculty of Veterinary Medicine, Chiang Mai University, Chiang Mai, Thailand; ^6^Center of Excellence in Veterinary Biosciences, Faculty of Veterinary Medicine, Chiang Mai University, Chiang Mai, Thailand

**Keywords:** polyunsaturated fatty acid EAB-277^®^, heart rate variability, tracheal collapse, inflammatory marker, malondialdehyde, dog

## Abstract

Canine tracheal collapse is a progressive disease in small breed dogs resulting from chronic inflammation of the tracheal mucosal lining. Polyunsaturated fatty acid EAB-277^®^ is one of the nutraceuticals that can alleviate inflammation and oxidative stress. Heart rate variability (HRV) is a prognostic tool related to sympathovagal balance and oxidative stress level, which is widely used with cardiorespiratory diseases. However, the effect of EAB-277^®^ on HRV in tracheal collapse dogs has rarely been investigated. In this study, 26 tracheal collapse dogs were divided into two groups. In the control group, the dogs received the standard treatment, whereas the dogs in the EAB-277^®^ group received standard treatment combined with EAB-277^®^. After being treated for 5 weeks, changes in radiographic findings, blood profiles, serum malondialdehyde, inflammatory markers, and HRV were evaluated. This study found that clinical signs were improved in both groups (*p* < 0.05). However, serum malondialdehyde (MDA), Interleukin-6 (IL-6), and Tumor necrosis factor-alpha (TNF-α) were decreased only in the EAB-277^®^ group after treatment for five weeks (*p* < 0.05) and the mean percent change of MDA, IL-6, and TNF-α at week five compared to baseline in the EAB-277^®^ group was greater than in the control group (*p* < 0.05). Additionally, greater sympathovagal imbalance indicated by decreased standard deviation of all normal R-R intervals (SDNN) and standard deviation of the averaged R-R intervals for all 5-minutes segments (SDANN) was found in the control group at week five compared to baseline (*P* < 0.05), whereas EAB-277^®^ improved SDNN and SDANN and decreased low frequency/high-frequency component (LF/HF ratio) after being treated for five weeks (*P* < 0.05). This study demonstrates that EAB-277^®^ improves clinical signs and attenuates HRV impairment by reducing oxidative stress and inflammation in tracheal collapse dogs.

## Introduction

Tracheal collapse is a common disease in small-breed dogs such as Pomeranians, Miniature and Toy Poodles, Yorkshire terriers, Chihuahuas, and Pugs (1). Usual clinical signs are respiratory distress, dyspnea, and harsh dry cough known as “goose-honking” which is often triggered by excitement and exercise (2). Tracheal collapse can be found in the cervical trachea and/or thoracic trachea depending on the severity (3) and is associated with the softening of hyaline cartilage rings due to a reduction of glycosaminoglycan, glycoprotein and chondroitin sulfate causing weakness and dorsoventral flattening of the tracheal rings resulting in injury to the mucosal lining of the trachea (3, 4). Moreover, in the chronic inflammatory process and mucosal change, epithelial squamous metaplasia can be found, which results in ciliary clearance dysfunction and generate coughing (1, 3).

A previous study demonstrated that the neuroendocrine pathway, the sympathetic (SNS) and the parasympathetic nervous system (PNS) are potent modulators of inflammation (5). The sympathetic nervous system can regulate both pro-inflammatory and anti-inflammatory processes by inducing the production of cytokines, whereas the parasympathetic nervous system and vagal nerve play an essential role in attenuating the inflammatory process (6). In addition, a positive correlation between oxidative stress and the inflammatory process has been reported (7, 8). Many inflammatory stimuli can induce excessive reactive oxygen species (ROS), resulting in the synthesis and secretion of pro-inflammatory cytokines such as TNF-α that play a critical role in the inflammatory process and can result in several chronic diseases (8, 9).

Heart rate variability (HRV) is a prognostic tool for evaluating sympathovagal balance and oxidative stress level (10). It has been used to evaluate the balance of the sympathetic and parasympathetic nervous systems by analyzing the variation in the beat-to-beat timing of the heart rate using portable electrocardiogram equipment or a Holter machine (11, 12). HRV has also been used as a prognostic tool for many diseases such as cardiovascular, respiratory, kidney, and neurological diseases (13–17). In addition, it can be used to monitor health status and adaptability as well as functionality related health status (5, 18). In veterinary medicine, Holter monitoring is a simple and non-invasive test that can serve many purposes, not only in cardiorespiratory disease but also in kidney diseases (19, 20). Moreover, HRV is a well-established and reliable index of cardiac vagal regulation which is inversely related to inflammatory markers and oxidative stress (6). Unfortunately, the use of HRV as a prognostic tool in tracheal collapse dogs has rarely been investigated.

Establishing priorities for tracheal collapse treatment depends on the severity of the tracheal collapse as well as other clinical signs (3). Medical management can include antitussive therapy, antibiotics, bronchodilators, and/or anti-inflammation medication, e.g., corticosteroids and NSAIDs) (21, 22). Many dogs respond to this medical therapy, but some have complications, especially with long-term use of corticosteroids which increases the risk of secondary bacterial infection, increases the respiratory rate, and induces weight loss that worsens the patient's clinical signs (3, 23). Polyunsaturated fatty acid from Green-Lipped Mussel Blend is a safe, natural product with high antioxidant and anti-inflammation performance which is widely used to treat several diseases including as osteoarthritis in both humans and animals (24–26).

EAB-277^®^ is a patented nutraceutical marine-based fatty acid-containing lipid fractions from the New Zealand green-lipped mussel combined with high phospholipids extracted from krill oil (Pharmalink International Co., Ltd.,). It is composed of 91 fatty acids, including omega 3 fatty acid, docosahexaenoic acid (DHA), eicosapentaenoic acid (EPA), docosapentaenoic acid (DPA), and eicosatetraenoic acid (ETA) as the key components. The patented component, EAB-277^®^, has been shown to have an anti-inflammatory effect by inhibiting the COX-2 and 5-lipoxygenase (LOX) pathways resulting in a reduction in pro-inflammatory cytokine production (27, 28). Moreover, in recent studies conducted in patients with the respiratory disease, it has been found to exert a beneficial effect on asthma by reducing the inflammatory process and attenuating hyperpnea (29). In contrast, another study reported that supplementation with polyunsaturated fatty acid from Green-Lipped Mussel blend did not improve pulmonary or respiratory muscle function in non-asthmatic elite runners (24).

However, in veterinary medicine, the beneficial effects of polyunsaturated fatty acid from the Green-Lipped Mussel blend related to HRV have rarely been investigated in tracheal collapse dogs. This study aims to investigate the effect of polyunsaturated fatty acid from Green-Lipped Mussel blend (EAB-277^®^) supplementation combined with standard therapy on physical examination parameters, radiographic findings, blood profile, oxidative stress, inflammatory markers, and HRV hypothesizing that EAB-277^®^ supplementation could improve clinical outcomes and reduce HRV impairment in tracheal collapse dogs. The findings obtained from this research help explain the effects of polyunsaturated fatty acid EAB-277^®^ from the Green-Lipped Mussel blend on the autonomic nervous system in tracheal collapse dogs and may also explain some of the beneficial and adverse effects of this nutraceutical on the tracheal collapse condition.

## Materials and Methods

### Animal Model and Research Protocol

In this study, tracheal collapse dogs who visited the Cardiopulmonary Clinic, Chiang Mai University's Small Animal Teaching Hospital received general screening tests, including history taking, physical examination, cardiovascular disease evaluation using echocardiography, chest X-ray, and blood collection. All tracheal collapse dogs have been treated and stabilized with standard therapy for 3 months before enrolling in the experiment. A nine-point body condition score (BCS) system was used. The same investigator allocated all scores. Dogs with a BCS of 7–9 were considered obese, whereas BCS of 4–5 were considered to be normal weight. Tracheal collapse dogs with symptomatic systemic diseases, metabolic diseases, cardiovascular diseases, chronic inflammatory diseases (e.g., orthopedic disease), periodontal disease, chronic kidney disease, cryptorchidism or tumors, or obesity were excluded from this study.

Twenty-six tracheal collapse small-breed dogs (13 males and 13 females, ages 2 to 18 years) were used in this study and divided into control and EAB-277^®^ groups. In the control group (*n* = 13), the dogs received only standard treatment for 5 weeks, whereas the EAB-277^®^ group (*n* = 13) was provided standard treatment combined with polyunsaturated fatty acid EAB-277^®^ from Green-Lipped Mussel blend for the 5 weeks. In this study, there were 6 breeds of dog including Pomeranian (*n* = 15), Chihuahua (*n* = 3), Poodle (*n* = 1), Pugs (*n* = 4), Shih Tzu (*n* = 2) and Yorkshire terrier (*n* = 1).

**Figure 1:**
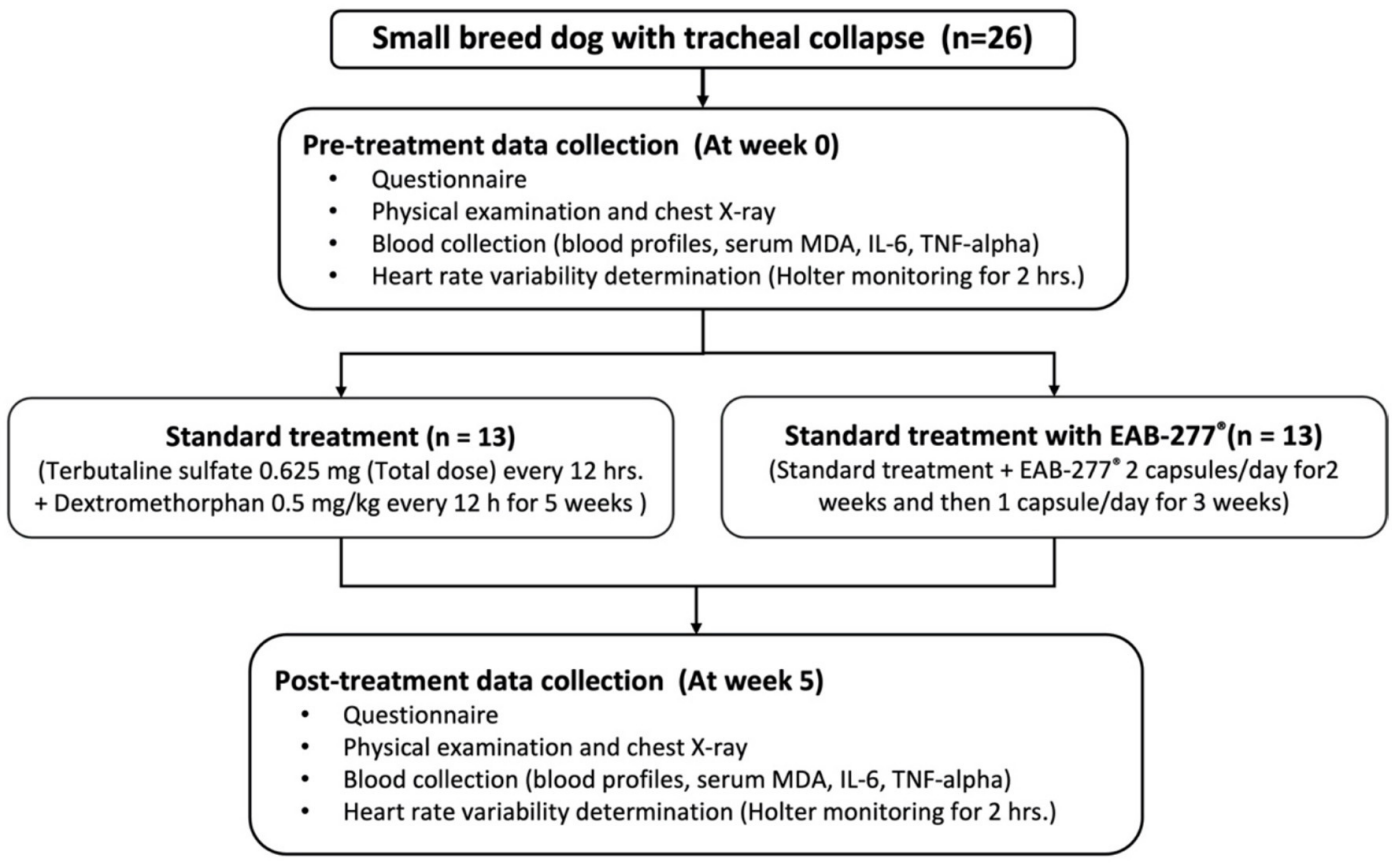
Schematic representation of the study protocol. At pre-treatment, all dogs underwent chest X-ray, blood collection, Holter recording for 2-h. Then, dogs were divided into two groups to receive the standard treatment or standard treatment combined with polyunsaturated fatty acid EAB-277^®^ from Green-Lipped Mussel blend for the 5 weeks. At post-treatment, all dogs underwent the re-assessment of chest X-ray, blood collection, Holter recording for 2-h to investigate cardiac sympathovagal balance, plasma oxidative stress, and inflammatory marker measurement. MDA, Malondialdehyde.

After being stabilized with standard therapy for 3 months, all dogs underwent chest X-ray, blood collection, Holter recording for 2-hours to investigate cardiac sympathovagal balance, plasma oxidative stress, and inflammatory marker measurement at baseline or pre-treatment (before being supplemented with EAB-277^®^) and re-evaluated these parameters at post-treatment after being supplemented with EAB-277^®^ for 5 weeks. The study was performed at the Small Animal Teaching Hospital, Faculty of Veterinary Medicine, Chiang Mai University. All owners signed an informed consent form, and all experiments were approved by the Ethics Committee of the Laboratory Animal Center, Faculty of Veterinary Medicine, Chiang Mai University (Ethical Number: R29/2562) (Figure 1). Written informed consent was obtained from the owners for the participation of their animals in this study.

### Questionnaire for Clinical Sign Determination

All the tracheal collapse dogs were evaluated pre-and post-treatment for clinical signs using an owner-reported questionnaire consisting of four series of questions using a rubric scoring system (cough symptoms, duration of coughing each time, activity-induced coughing, frequency of showing cough symptoms or choking after eating or drinking) with a range of scores of 0 to 2 as shown in Table 1.

**Table 1:** Representative questionnaire in rubric scoring system for clinical sign determination.

**Clinical signs**	**Score 0**	**Score 1**	**Score 2**
Coughing frequency	≤ 3 times/day	3–7 times/day	≥7 times/day
Coughing duration	≤ 10 s	11–60 s	≥60 s
Activity-induced coughing	Sometimes cough due to exercise, restrain, or excitement	Always cough due to exercise, restrain, or excitement	Always cough without exercise, restrain, or excitement
Coughing during eating or drinking	≤ 2 times/week	2–7 times/week	≥7 times/week

### Physical Examination and Chest X-Ray

All dogs were evaluated for standard health status by physical examination (inspection, palpation, percussion, and auscultation), including general appearance and vital signs such as mucous membranes, capillary refill time, rectal temperature, heart rate, heart sounds, pulse rate, and hydration status. A chest X-ray was performed for evaluating tracheal diameter/thoracic inlet distance ratio (TD/TI ratio). Vertebral heart scale (VHS) and vertebral left atrial side (VLAS) were also evaluated.

### Standard Treatment and Polyunsaturated Fatty Acid EAB-277^®^ Dosages

The standard treatment dogs (*n* = 13) received terbutaline sulfate (President Inter Pharma Co., Ltd., Thailand) 0.625 mg every 12 hrs. (total dose/day) and dextromethorphan (Greater Pharma Manufacturing Co., Ltd., Thailand) 0.5 mg/kg every 12 h for 5 weeks (21). The dogs in the EAB-277^®^ group (*n* = 13) were prescribed the same standard treatment plus two capsules (loading dose) of polyunsaturated fatty acid EAB-277^®^ from the Green-Lipped Mussel blend at 8.00 AM for the first 2 weeks, followed by one capsule for the final 3 weeks. The dosage of EAB-277^®^ has been chosen based on the recommendation from the manufacturer, whereas the total duration of treatment for both groups was 5 weeks (30).

### Blood Profiles Determination

Blood was collected by venipuncture and divided into three fractions at baseline (pre-treatment) and week 5 (post-treatment). The first fraction was placed in tubes containing potassium ethylene diamine tetra-acetic acid (EDTA) for complete blood count (CBC) analysis. The second fraction was placed in tubes with silica dioxide (a clot activator) for blood chemistry analysis [blood urea nitrogen (BUN), creatinine, alanine aminotransferase (ALT), alkaline phosphatase (ALP), total protein (TP), albumin (ALB) and globulin (GLB)]. The third fraction was prepared from clotting blood by centrifugation at 1,500 x g at 4°C for 15 min and then stored at −80 °C for further MDA, TNF-α, and IL-6 analysis.

### Malondialdehyde (MDA) and Inflammatory Cytokine (TNF-α, IL-6) Level Determination

Serum MDA levels were analyzed by measuring the MDA concentration in the serum as thiobarbituric acid (TBA) using high-performance liquid chromatography (HPLC) based assay. Plasma or serum MDA was mixed with 0.44 M H_3_PO_4_ and 0.6% TBA solution, resulting in the generation of pink-colored products called thiobarbituric acid reactive substances (TBARS). Serum TBARS concentrations were determined directly from a standard curve and reported as an MDA equivalent concentration using an HPLC-based assay (31, 32). In addition, canine serum inflammatory cytokine, including TNF-α and IL-6, were assessed by direct enzyme-linked immunosorbent assay (ELISA) according to the manufacturer's instructions (Sigma-Aldrich, Saint Louis, MO, USA).

### Heart Rate Variability Determination

Holter monitoring was investigated by an experienced cardiologist with randomized and blinded techniques at baselines (pre-treatment) and post-treatment. Data from a Holter machine was collected in the morning (8.00–12.00 AM). The dogs were connected to the Holter machine (SEER™ 1000, GE Healthcare, Waukesha, WI, USA) by standard chest leads for 3 h while in a quiet environment in a separate room. After recording, the ECG waveform was automatically evaluated using a MAR^TM^ program, followed by manual removal of ECG artifacts and atrial or ventricular premature complexes. After that, a continuous 120 min (2 h) ECG segment was selected and analyzed using the MAR^TM^ program. Power spectra of RR interval variability were obtained using the fast Fourier transform (FFT) algorithm and the three major oscillatory components, including high frequency (HF) (0.15–0.4 Hz), low frequency (LF) (0.04–0.15 Hz), and very low frequency (VLF) (0.003–0.04 Hz), were determined (33). The parasympathetic activity was regarded as HF, whereas sympathetic and parasympathetic activity associations were regarded as LF (34, 35). The LF/HF ratio was considered an index of cardiac sympathovagal tone balance (36, 37). An increased LF/HF ratio indicates a cardiac sympathovagal imbalance.

### Statistical Analysis

All data are expressed as mean ± SEM. Statistical comparison within groups (week 1 and week 5 for each group) was analyzed using the paired *t*-test when the measurements showed a normal distribution and the Wilcoxon matched-pairs signed-rank test when they showed a non-normal distribution. Results from the questionnaire were analyzed using the Kruskal-Wallis test. All statistical analyses were performed with commercial software for Windows. Statistical significance was set at the level of *P*-values <0.05.

## Results

### Both Standard Treatment Alone and Standard Treatment Combined With EAB-277^®^ Improved All Clinical Signs as Evaluated by Questionnaire

**Figure 2:**
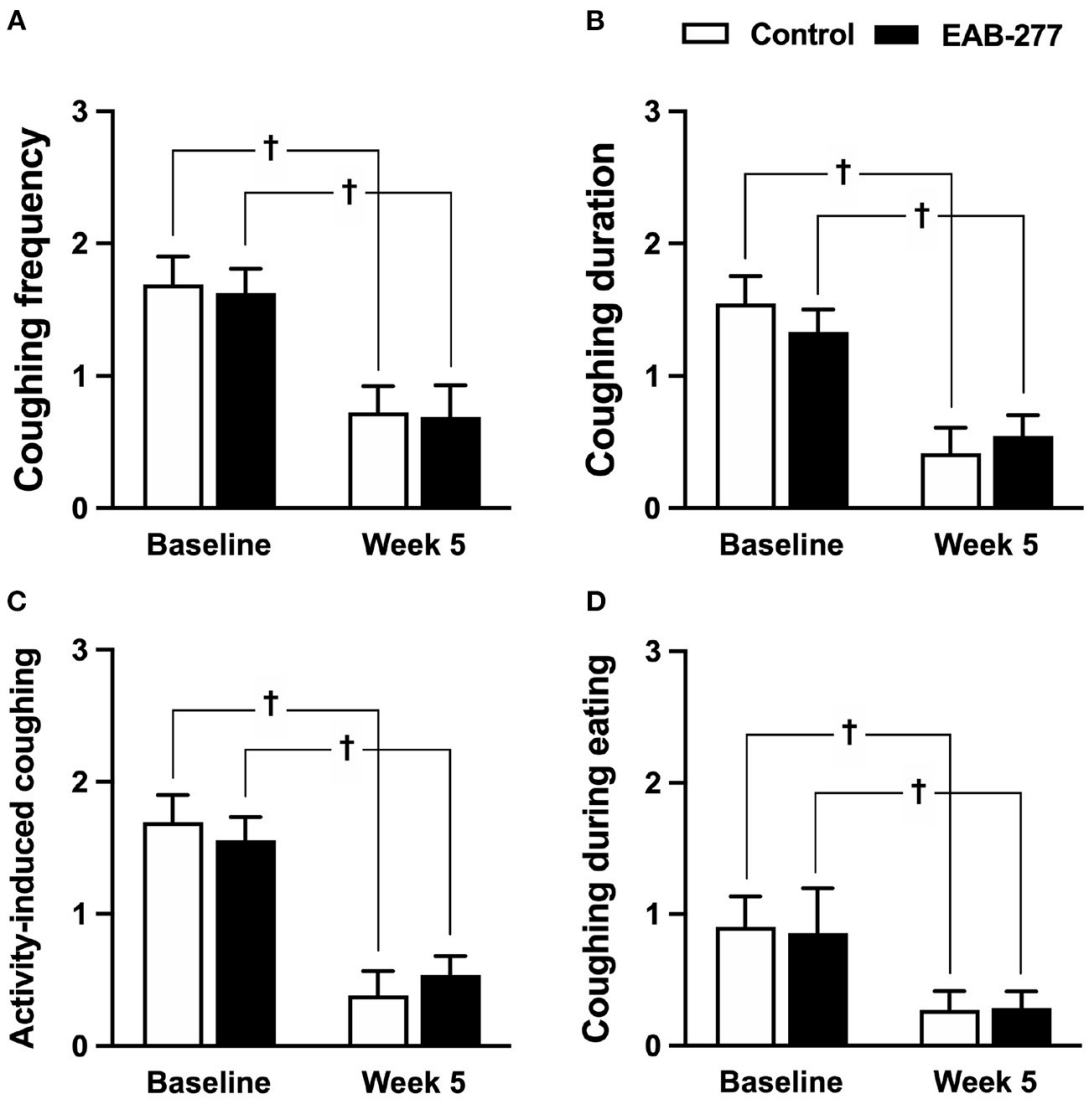
Clinical signs of tracheal collapse at baseline and at week 5 were evaluated by questionnaire in the control and EAB-277^®^ groups. All clinical signs, **(A)** coughing frequency, **(B)** coughing duration, **(C)** activity-induced coughing, and **(D)** coughing during eating, at baseline were not different between the groups. After treatment for 5 weeks, the clinical signs as evaluated by questionnaire were improved in both the control group and the EAB-277^®^ group compared to baseline in the same group. ^†^*P* ≤ 0.05 compared to baseline in the same group.

There were no statistical differences between the control and EAB-277^®^ groups for all clinical signs of tracheal collapse as indicated by the questionnaire at baseline (*p* > 0.05) (Figures 2A–D). Moreover, the results from the questionnaire, including coughing frequency, coughing duration, activity-induced coughing and coughing during eating, demonstrated that both the standard treatment (control groups) and standard treatment combined with EAB-277^®^ (EAB-277^®^ group) for 5 weeks could decrease all clinical signs evaluated by questionnaire compared to baseline in the same group (p ≤ 0.05) (Figures 2A–D). These questionnaire results suggest that EAB-277^®^ supplementation together with standard treatment can improve clinical signs the same as a standard treatment alone in tracheal collapse dogs.

### EAB-277^®^ Supplementation With Standard Treatment for 5 Weeks Did Not Have an Adverse Effect on the Physical Examination Parameters in Tracheal Collapse Dogs

Physical examination, including heart rate, respiratory rate, and temperature, showed no statistical difference (*p* > 0.05) between the standard treatment group and the standard treatment plus the EAB-277^®^ group (Table 2). This suggests that EAB-277^®^ supplementation in tracheal collapse dogs does not show adverse effects on body condition score (BCS), heart rate, respiratory rate, or body temperature.

**Table 2:** Representative general information and physical examination parameters in tracheal collapse dogs.

**Parameter**	**Standard treatment (*n =* 13)**		**Standard treatment with EAB-277^^®^^ group (*n =* 13)**	
	**Baseline**	**Week 5**	**Baseline**	**Week 5**
Age (year)	7.86 ± 1.20	7.86 ± 1.20	6.86 ± 1.04	6.86 ± 1.04
BCS (9 scores system)	5.93 ± 0.25	5.93 ± 0.25	6.07 ± 0.32	6.07 ± 0.32
Body temperature (F)	101.37 ± 0.23	101.49 ± 0.18	101.05 ± 0.18	101.38 ± 0.20
Heart rate (bpm)	109.71 ± 0.69	108.50± 0.67	109.86 ± 0.91	109.14 ± 0.93
Respiratory rate (bpm)	53.29 ± 0.97	51.71 ± 0.96	59.43 ± 0.92	60.57 ± 0.99

*All values are presented as mean + SEM. BCS, body condition score; bpm, beats per minute; tpm, time per minute; °F, degrees Fahrenheit*.

### EAB-277^®^ Supplementation Plus Standard Treatment for 5 Weeks Did Not Show Adverse Effects on the Blood Profile Parameters of Tracheal Collapse Dogs

There was no difference in any of the blood profile parameters between baseline and week 5 in the same group (*p* > 0.05). All blood parameters were in the normal range, which suggests that EAB-277^®^ supplementation in tracheal collapse dogs does not have an adverse effect on blood profile parameters (Table 3).

**Table 3:** Effect of standard treatment and EAB-277^®^ on blood morphology and blood chemistry profile in tracheal collapse dogs.

**Parameter**	**Standard treatment (*n =* 13)**		**Standard treatment plus EAB-277^^®^^ (*n =* 13)**		**References (38)**
	**Baseline**	**Week 5**	**Baseline**	**Week 5**	
**Blood morphology**					
Hct (%)	47.67 ± 1.41	48.20 ± 1.03	49.00 ± 1.10	46.50 ± 0.34	35–57
Hgb (g/dl)	16.33 ± 0.46	16.19 ± 0.37	17.60 ± 0.51	15.98 ± 0.24	11.9–18.1
RBCs (x10^6^/μL)	6.50 ± 0.27	6.89 ± 0.12	6.79 ± 0.33	6.63 ± 0.17	4.95–7.87
MCV (fl)	71.68 ± 0.88	70.95 ± 0.83	69.62 ± 0.53	67.73 ± 0.69	60–77
MCH (pg)	23.82 ± 0.23	23.65 ± 0.27	24.28 ± 0.28	23.72 ± 0.27	21–26.2
MCHC (g/dl)	33.36 ± 0.29	33.48 ± 0.26	34.88 ± 0.32	34.00 ± 0.24	32–36.3
WBC (x10^3^/μL)	12.37 ± 0.80	11.14 ± 0.50	11.14 ± 0.50	10.98 ± 0.60	5–14.1
Neu (band) (x10^3^/μL)	0.00 ± 0.00	0.02 ± 0.02	0.02 ± 0.02	0.05 ± 0.05	0–4.5
Neu (seg) (x10^3^/μL)	7.06 ± 0.77	5.90 ± 0.52	7.15 ± 0.69	8.21 ± 0.27	2.9–12
Lym(x10^3^/μL)	2.02 ± 0.30	1.75 ± 0.28	1.49 ± 0.14	1.66 ± 0.19	0.4–2.9
Mon (x10^3^/μL)	0.42 ± 0.08	0.53 ± 0.07	0.53 ± 0.09	0.60 ± 0.10	0.1–1.4
Eos (x10^3^/μL)	0.39 ± 0.07	0.51 ± 0.10	0.45 ± 0.09	0.57 ± 0.09	0–1.3
Bas (x10^3^/μL)	0.01 ± 0.01	0.00 ± 0.00	0.03 ± 0.01	0.00 ± 0.00	0–1.4
Platelet count (x10^3^/μL)	378.00 ± 33.82	383.18 ± 24.39	296.00 ± 22.12	371.00 ± 20.84	211–621
**Blood chemistry profiles**					
BUN (mg/dl)	12.13 ± 0.99	15.46 ± 1.57	17.01 ± 1.31	17.19 ± 1.66	8–28
Cre (mg/dl)	0.94 ± 0.05	0.94 ± 0.06	0.97 ± 0.06	1.00 ± 0.03	0.5–1.7
ALT (U/L)	52.91 ± 8.10	55.67 ± 11.61	69.33 ± 11.95	54.45 ± 6.88	10–109
ALP (U/L)	87.50 ± 14.20	85.30 ± 13.58	72.00 ± 15.19	56.55 ± 9.91	1–114
TP (g/dl)	7.25 ± 0.20	7.26 ± 0.13	7.12 ± 0.21	7.22 ± 0.15	5.4 −7.5
ALB (g/dl)	3.18 ± 0.11	3.03 ± 0.09	3.20 ± 0.10	3.06 ± 0.13	2.3–3.1
GLB (g/dl)	4.08 ± 0.20	3.92 ± 0.17	4.01 ± 0.19	3.88 ± 0.18	2.7–4.4

*All values are presented as mean + SEM. Hct, hematocrit; Hgb, hemoglobins; RBCs, red blood cells; MCV, mean corpuscular volume; MCH, mean corpuscular hemoglobin; MCHC, mean corpuscular hemoglobin concentration; WBC, white blood cells; Neu (band), Neutrophil band; Neu (seg), Neutrophil segment; Lym, Lymphocyte; Mon, monocyte; Eos, eosinophil; Bas, basophil; PLT, platelet count; BUN, blood urea nitrogen; Cre, creatinine; ALT, alanine aminotransferase; ALP, alkaline phosphatase; TP, total protein; ALB, albumin; GLB, globulin*.

### EAB-277^®^ Supplementation Plus Standard Treatment for 5 Weeks Did Not Improve Tracheal or Cardiac Structure as Evaluated by Chest X-Ray in Tracheal Collapse Dogs

Assessment of the trachea's size was made by measuring the tracheal diameter to thoracic inlet distance (TD: TI) ratio, while the heart size and left atrium size were evaluated by measuring the vertebral heart score (VHS) and the vertebral left atrial size (VLAS) from a chest x-ray, respectively. In this study, all dogs demonstrated tracheal hypoplasia indicated by a TD: TI ratio below the reference range. Moreover, VHS and VLAS were normal, and none of the tracheal collapse dogs had the cardiovascular disease, as confirmed by chest x-ray. Interestingly, none of the parameters evaluated using chest X-rays (TD: TI ratio, VHS, VLAS) at week 5 were significantly different when compared to baseline in the same group (*p* > 0.05). This suggests that EAB-277^®^ does not improve the cardiac and tracheal structure as evaluated by chest X-ray in tracheal collapse dogs (Table 4).

**Table 4:** Radiography parameters of standard treatment and EAB-277^®^ groups at baseline and at week 5.

**Parameter**	**Standard treatment (*n =* 13)**		**Standard treatment with EAB-277^^®^^ (*n =* 13)**		**References**
	**Baseline**	**Week 5**	**Baseline**	**Week 5**	
TD:TI ratio	0.13 ± 0.03	0.13 ± 0.03	0.13 ± 0.05	0.14 ± 0.06	<0.16 (39)
VHS	10.46 ± 0.63	10.50 ± 0.58	10.20 ± 0.52	10.51 ± 1.06	9.5–10.7 (40)
VLAS	1.92 ± 0.33	1.91 ± 0.33	2.04 ± 0.38	1.98 ± 0.29	1.4–2.2 (41)

*All values are presented as mean + SEM. TD:TI ratio, ratio of the tracheal lumen diameter at the thoracic inlet to the thoracic inlet diameter; VHS, Vertebral Heart Score; VLAS, Vertebral Left Atrial Score*.

### EAB-277^®^ Supplementation With Standard Treatment for 5 Weeks Can Decrease Serum Malondialdehyde (MDA) in Tracheal Collapse Dogs

**Figure 3:**
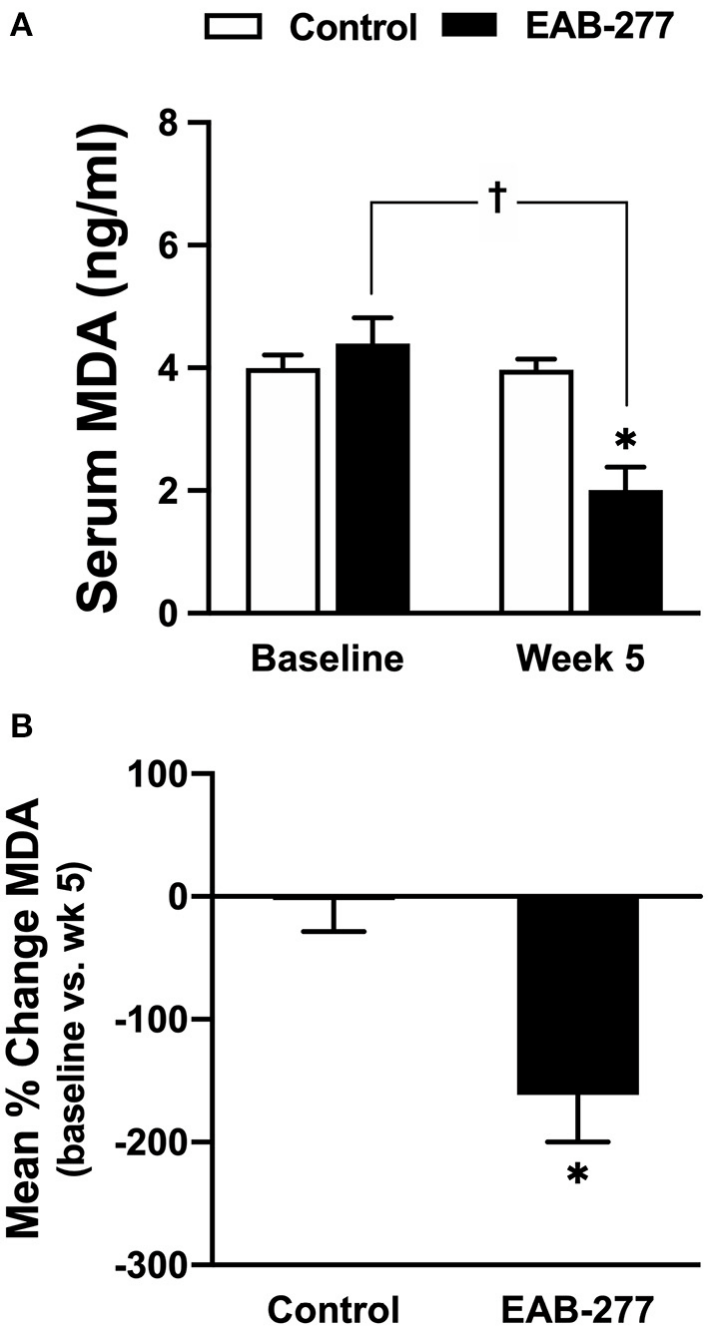
Effect of a standard treatment plus EAB-277^®^ on serum MDA levels in tracheal collapse dogs. Serum MDA level before and after treatment was not different in the control group, whereas serum MDA level was decreased at week 5 in the treatment group compared to the control group and compared to the treatment group at baseline **(A)**. Moreover, the mean percent change of serum MDA at week 5 in the treatment group was greater than the mean percent change in the control group **(B)**. *P ≤ 0.05 compared to the control group. ^†^*P* ≤ 0.05 compared to baseline in the same group.

An oxidative stress marker was used to assess MDA level using a high-performance liquid chromatography (HPLC) system. At baseline, the serum MDA was not different between the control and EAB-277^®^ groups (Figure 3A). After treatment for 5 weeks, the serum MDA level in the control group (standard treatment without EAB-277^®^ supplementation) was not different from baseline (Figure 3A) (*p* > 0.05), whereas the serum MDA level in the EAB-277^®^ group (standard treatment plus EAB-277^®^ supplementation) was significantly decreased when compared to baseline in the same group (*p* ≤ 0.05) as shown in Figure 3A. Moreover, the mean percent change of serum MDA at week 5 compared to baseline in the treatment group (62.7 ± 87.5%) was significantly greater than the mean percent change in the control group (2 ± 25.8%) (*p* ≤ 0.05) (Figure 3B). This suggests that EAB-277^®^ supplementation plus standard treatment can reduce oxidative stress production in tracheal collapse dogs.

### EAB-277^®^ Supplementation Plus Standard Treatment for 5 Weeks Can Decrease Canine Serum IL-6 and Canine TNF-α in Tracheal Collapse Dogs

**Figure 4:**
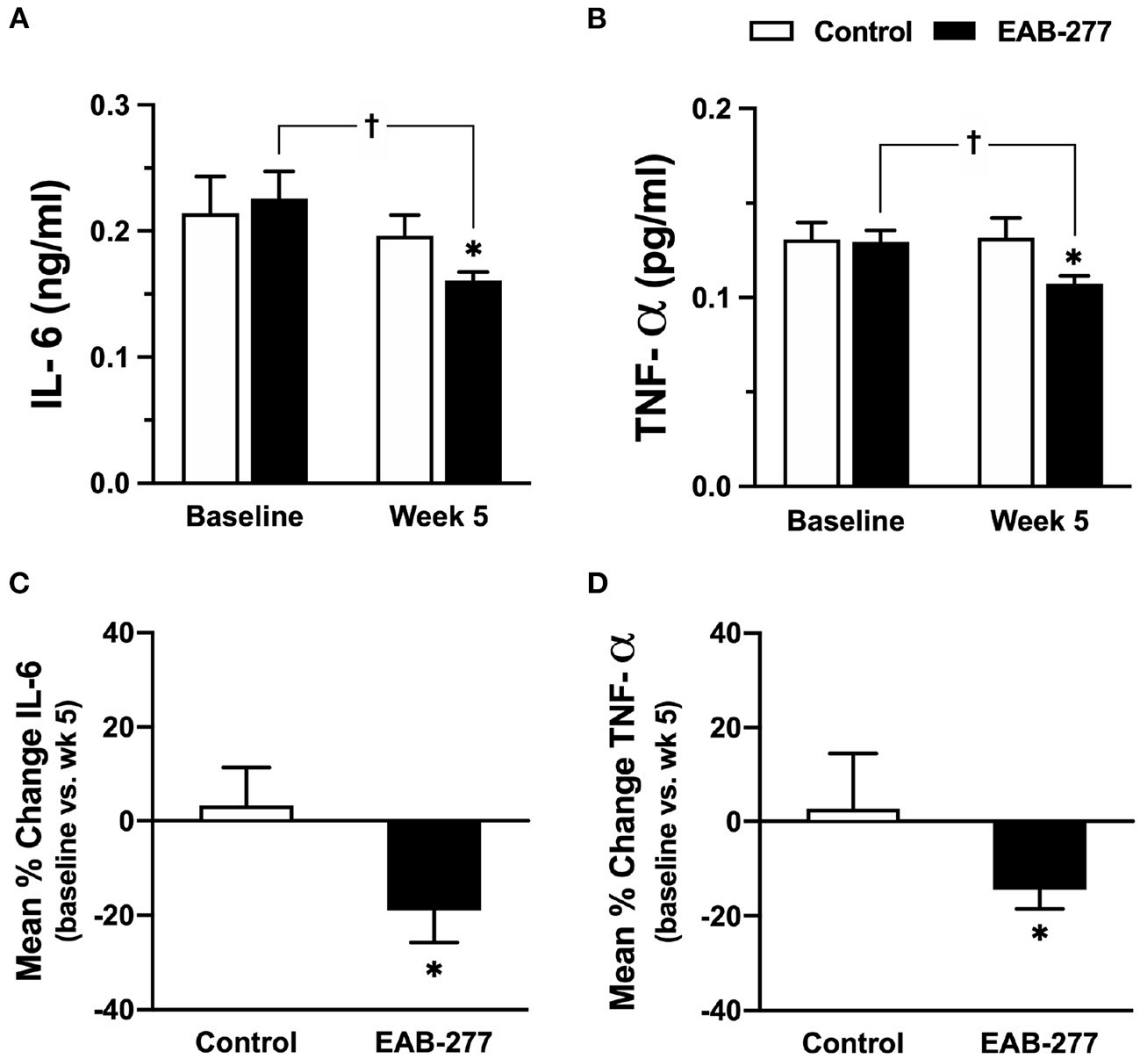
Effect of standard treatment and EAB-277^®^ on serum IL-6 and serum TNF-α in tracheal collapse dogs. **(A)** Serum IL-6 and **(B)** TNF-α were not different between groups at baseline, whereas serum IL-6 and TNF-α were decreased at week 5 in the EAB-277^®^ group compared to baseline in the same group. Moreover, the mean percent change of serum IL-6 and TNF-α at week 5 in the treatment group was greater than the control group **(C,D)**. *P ≤ 0.05 compared to the control group. ^†^*P* ≤ 0.05 compared to baseline in the same group.

Canine serum inflammatory cytokine (canine IL-6 and canine TNF-α) were assessed by ELISA kit (Sigma-Aldrich, Saint Louis, MO, USA). At baseline, serum canine IL-6 and TNF-α were not significantly different between the control and EAB-277^®^ groups (Figures 4A,B). Interestingly, after treatment for 5 weeks, serum canine IL-6 and TNF-α in the EAB-277^®^ group was significantly decreased when compared to baseline in the same group, whereas these inflammatory markers in the control group were not different compared to baseline in the same group (Figures 4A,B). Moreover, serum canine IL-6 and TNF-α in the EAB-277^®^ group was significantly lower than the control group at week 5 (Figures 4A,B). In addition, the mean percent change of serum IL-6 and TNF-α at week 5 compared to week 1 baseline in the treatment group (IL-6; 19 ± 6.7% and TNF-α; 13 ± 3.6%) were significantly greater than the mean percent change in the control group (IL-6; 3.2 ± 8.2% and TNF-α; 1.5 ± 10.4%) (p ≤ 0.05) (Figures 4C,D). This suggests that EAB-277^®^ supplementation plus standard treatment can reduce serum inflammatory cytokine production in tracheal collapse dogs.

### EAB-277^®^ Supplementation Plus Standard Treatment for 5 Weeks Can Attenuate Sympathovagal Imbalance in Tracheal Collapse Dogs

**Figure 5:**
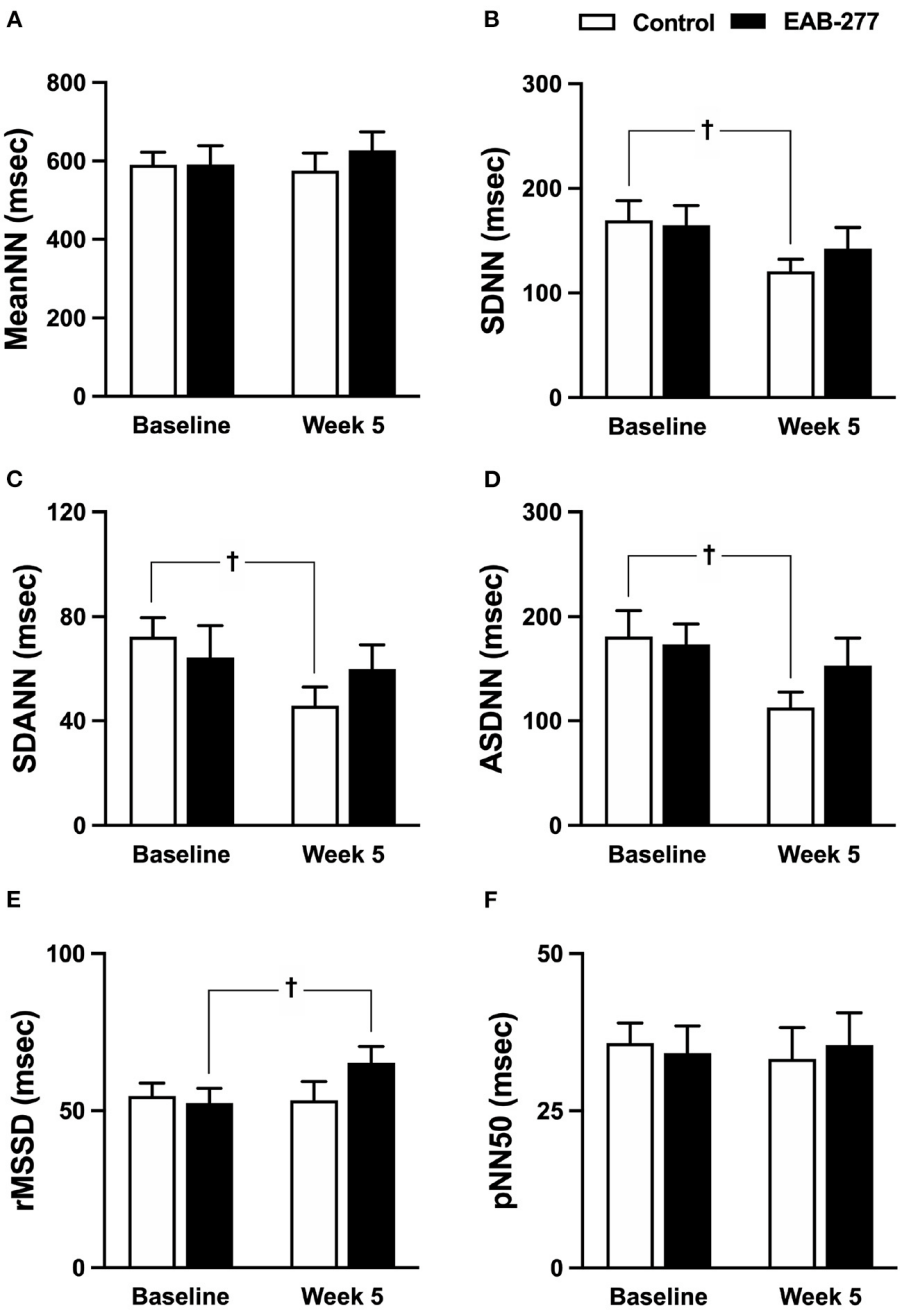
Effect of standard treatment and EAB-277^®^ on time-domain heart rate variability in tracheal collapse dogs. Time-domain HRV demonstrated that **(A)** mean NN, **(B)** SDNN, **(C)** SDANN, **(D)** ASDNN, **(E)** rMSSD, and **(F)** pNN50 were not different in the two groups at baseline. In the control group, SDNN, SDANN, and ASDNN were decreased at week 5 compared to baseline. In the EAB-277^®^ group, **(E)** rMSSD was increased at week 5 compared to baseline, whereas mean NN, SDNN, SDANN, ASDNN, and pNN50 were not different before and after treatment. Mean NN, mean of N-N intervals; SDNN, standard deviation of all normal R-R intervals; SDANN, standard deviation of the averaged N-N intervals for all 5-min segments; ASDNN, average of the 5-min standard deviations of N-N intervals; rMSSD, Square root of the mean of successive N-N intervals differences and pNN5, the percentage of successive N-N intervals > 50 msec. ^†^*P* ≤ 0.05 compared to baseline in the same group.

**Figure 6:**
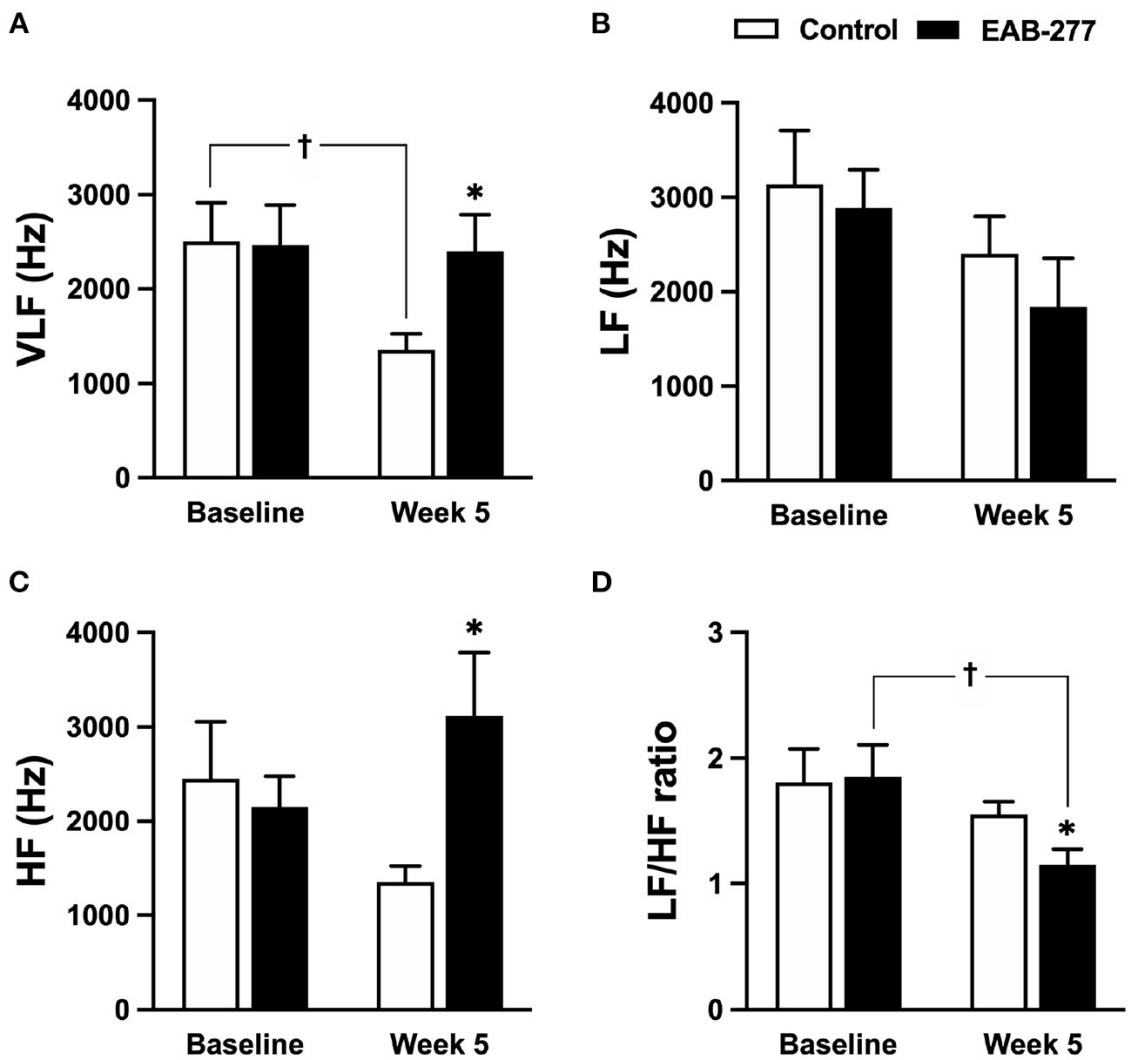
Effect of standard treatment and EAB-277^®^ on frequency-domain heart rate variability in tracheal collapse dogs. Frequency-domain HRV demonstrated that **(A)** VLF, **(B)** LF, **(C)** HF, and **(D)** LF/HF ratios were not different between groups at baseline. At week five, frequency-domain HRV in the control group **(A–D)** showed that **(A)** VLF parameter was decreased compared to baseline, whereas **(B)** LF, **(C)** HF, and **(D)** LF/HF ratio at week 5 were not different from baseline. In the EAB-277^®^ group, **(C)** the HF parameter was increased at week 5 compared to the control group, and **(D)** the LF/HF ratio at week 5 was decreased compared to baseline, whereas **(A)** VLF, **(B)** LF, and **(C)** HF were not different before and after treatment. VLF, Very low-frequency component; LF, Low frequency component; HF, High frequency component. LF/HF ratio, Low frequency/high-frequency component. *P ≤ 0.05 compared to baseline in the same group. ^†^*P* ≤ 0.05 compared to baseline in the same group.

HRV parameters of the control group (standard treatment) and the EAB-277^®^ group (standard treatment with the EAB-277^®^) were investigated at baseline and week five. Comparison of HRV parameters are shown in the time and frequency domains presented in Figures 5, 6, respectively.

In the time domain HRV, the variation of the RR interval indicated by SDNN, SDANN, and ASDNN was significantly decreased (*p* ≤ 0.05) in the control group at week 5 compared to baseline (Figures 5A–F), whereas other time-domain parameters in the control group before and after treatment were not significantly different between time courses (Figures 5A,E,F). Interestingly, SDNN, SDANN, and ASDNN in the EAB-277^®^ group were not significantly different before and after supplementation with EAB-277^®^ for 5 weeks (Figures 5A–F). Moreover, the parasympathetic tone indicated by rMSSD in the EAB-277^®^ group was significantly increased at week 5 compared to baseline in the same group (Figure 5E).

In the frequency-domain HRV, the parameters were not significantly different between the control and EAB-277^®^ groups at baseline (Figures 6A–D). After treatment for 5 weeks, tracheal collapse dogs in the control group had decreased parasympathetic tone as indicated by VLF at week 5 compared to baseline in the same group and compared to the EAB-277^®^ group (*p* ≤ 0.05) (Figure 6A). Other parameters of the control group at week 5 were not significantly different among time courses from baseline values (*p* > 0.05) (Figures 6B–D). Interestingly, tracheal collapse dogs in the EAB-277^®^ group found an increase of improvement in parasympathetic tone as indicated by increased HF at week 5 compared to the control group (Figure 6C) and a decreased LF/HF ratio at week 5 compared to baseline and between the groups (Figure 6D). This suggests that standard therapy plus EAB-277^®^ supplementation for 5 weeks can improve sympathovagal imbalance by increasing parasympathetic activity in tracheal collapse dogs.

## Discussion

This study demonstrated the beneficial effects of EAB-277^®^ supplementation combined with standard therapy on heart rate variability in dogs with tracheal collapse. The major findings of this study are as follows. First, EAB-277^®^ supplementation for 5 weeks did not affect the physical examination results, radiographic findings, or blood profile parameters in the tracheal collapse dogs. Second, EAB-277^®^ supplementation for 5 weeks decreased oxidative stress and inflammatory marker compared to standard therapy as indicated by serum MDA, canine serum IL-6 and canine TNF-α in tracheal collapse dogs. Third, EAB-277^®^ supplementation for 5 weeks attenuated sympathovagal imbalance by increasing parasympathetic activity in tracheal collapse dogs. Fourth, using a questionnaire or evaluating TD/TI by chest X-ray was not accurate in evaluating the improvement of tracheal collapse in dogs.

Regarding clinical signs evaluated by questionnaire, this study found improvement in clinical signs in tracheal collapse dogs after treatment compared to pre-treatment in both groups, including a decrease in the frequency and duration of coughing, coughing induced by activities such as exercise, and eating and drinking. A limitation of the questionnaire used in this study was the wide range of response scores and had much personal information, which could affect the reliability of the results. This suggests that a questionnaire might not be appropriate as a prognostic tool for routine follow-up and routine examination in tracheal collapse dogs.

Moreover, this study demonstrated that EAB-277^®^ supplementation did not show either beneficial or adverse effects on blood profile parameters, which is consistent with previous studies in the dog and human models (25, 42). A previous study in dogs that included PCSO-524^®^ combined with an anti-inflammatory drug for 4 weeks demonstrated that PCSO-524^®^ supplementation alone does not affect blood profiles or physical examination parameters (25). Moreover, a study in humans demonstrated that PCSO-524^®^ supplementation for 12 weeks does not affect the liver function or blood profiles in humans with osteoarthritis (42). Interestingly, although ALP and ALT parameters in EAB-277^®^ supplementation combined with standard therapy tend to be reduced after treatment for 5 weeks, there was no statistically significant difference compared to baseline in the EAB-277^®^ group. A larger sample is needed to confirm these findings, as is further study regarding the beneficial effect on liver function.

In addition, EAB-277^®^ supplementation did not show either beneficial or adverse effects on the TD/TI ratio as evaluated by chest X-ray in tracheal collapse dogs. This is consistent with a previous study that suggested that 8% of dogs with tracheal collapse could not be accurately assessed using radiographic findings due to the radiographs being related to the respiratory phase (3). Moreover, radiographic findings usually underestimate the severity of the tracheal collapse, suggesting that bronchoscopy is a better technique for evaluating and grading the degree of tracheal collapse (3). However, bronchoscopy is not practical for routine follow-up and routine examination.

Time-domain HRV, especially the SDNN and rMSSD, could be used to determine parasympathetic nervous system dominance using a short duration recording (43, 44), whereas SDNN is significantly correlated with ASDNN and SDANN (45). Interestingly, the present study found that SDNN, SDANN, and ASDNN were significantly decreased in the standard treatment group at week five, implying a reduction of parasympathetic tone. On the other hand, these time-domain parameters (SDNN, SDANN, and ASDNN) were not changed after supplementation with EAB-277^®^ for 5 weeks, indicating that EAB-277^®^ might exert a beneficial effect on HRV by attenuating sympathovagal imbalance in tracheal collapse dogs. Moreover, the tracheal collapse dogs in the EAB-277^®^ group demonstrated an increase in the rMSSD parameter, implying an increase of parasympathetic tone in tracheal collapse dogs that received EAB-277^®^ supplementation for 5 weeks.

In frequency domain HRV, several studies have demonstrated that increasing the LF/HF ratio can determine the increase of sympathetic tone and can also indicate a sympathovagal imbalance (5, 32, 46, 47). Interestingly, frequency-domain HRV in the present study was consistent with the time-domain HRV and demonstrated that EAB-277^®^ supplementation significantly decreased the LF/HF ratio, implying that EAB-277^®^ supplementation for 5 weeks can improve the sympathovagal imbalance in tracheal collapse dogs. On the other hand, the standard group showed decreased parasympathetic activity as indicated by decreasing VLF parameters, while the LF/HF ratio in the standard group was not different at week 5 compared to baseline.

This study also demonstrated that EAB-277^®^ supplementation could reduce MDA, IL-6, and TNF-α levels in tracheal collapse dogs, while the standard treatment alone cannot. This is consistent with previous studies reporting a positive correlation between oxidative stress and inflammatory process (7, 8). The present study also demonstrated that tracheal collapse is a chronic inflammatory disease, which induces the synthesis and secretion of pro-inflammatory cytokines such as TNF-α and IL-6, resulting in excessive reactive oxygen species (ROS). Interestingly, a previous study reported that both time and frequency domain HRV parameters such as SDNN, rMSSD, and LF/HF ratio correlated inversely with MDA level (32, 33, 48, 49), which is consistent with the present study. Moreover, a previous study demonstrated that the rostral ventrolateral medulla (RVLM) of the brain stem region has an important role in controlling basal and reflex changes in sympathetic nervous system activity (50). Increasing oxidative stress has been shown to contribute to sympathoexcitation at the level of the RVLM in several animal models (50).

The present study suggests that EAB-277^®^ supplementation can attenuate sympathovagal imbalance by increasing parasympathetic activity in tracheal collapse dogs based on evaluating time-domain and frequency-domain HRV. A possible mechanism is the decrease of oxidative stress (MDA) and inflammatory cytokine (IL-6 and TNF-α) plus the resulting decrease in sympathetic activity and increases in parasympathetic activity via the rostral ventrolateral medulla (RVLM) axis. However, our study did demonstrate that radiographic studies and questionnaires have low accuracy for the evaluation of improvement of clinical signs. This study further suggests that HRV parameters could potentially be used as a prognostic physio-maker for assessing the inflammatory response indirectly in clinical evaluations of tracheal collapse dogs.

## Conclusions

Standard therapy supplemented with EAB-277^®^ can decrease MDA and inflammatory cytokine (IL-6 and TNF-α) levels, resulting in improved time domain and frequency domain HRV in tracheal collapse dogs. EAB-277^®^ supplementation can also attenuate clinical signs and increase the quality of life of tracheal collapse dogs after 5 weeks of treatment; however, further study is needed to confirm these findings.

## Data Availability Statement

The original contributions presented in the study are included in the article/supplementary material, further inquiries can be directed to the corresponding author.

## Ethics Statement

The animal study was reviewed and approved by Ethics Committee of the Laboratory Animal Center, Faculty of Veterinary Medicine, Chiang Mai University (Ethical Number: R29/2562). Written informed consent was obtained from the owners for the participation of their animals in this study.

## Author Contributions

WP and RM reviewed, edited, and wrote the manuscript. WP provided the experimental design, laboratory work, review, editing, and assisted in all aspects of producing the manuscript. All authors conducted the experiments, analyzed the statistical data, read, and approved the final version of this manuscript.

## Funding

This work was partially supported by Pharmalink International Limited and Chiang Mai University (CMU) (WP, RM, and CB), which provided the necessary budget through the Center of Excellence in Veterinary Biosciences, Faculty of Veterinary Medicine, Chiang Mai University, R000029941 (WP), and the Faculty of Veterinary Medicine, Chiang Mai University (WP, RM, and CB). The authors declare that this study received funding from Pharmalink International Limited. The funder was not involved in the study design, collection, analysis, interpretation of data, the writing of this article or the decision to submit it for publication.

## Conflict of Interest

The authors declare that the research was conducted in the absence of any commercial or financial relationships that could be construed as a potential conflict of interest.

## Publisher's Note

All claims expressed in this article are solely those of the authors and do not necessarily represent those of their affiliated organizations, or those of the publisher, the editors and the reviewers. Any product that may be evaluated in this article, or claim that may be made by its manufacturer, is not guaranteed or endorsed by the publisher.
