# Corrigendum: Polyunsaturated fatty acid EAB-277^®^ supplementation improved heart rate variability and clinical signs in tracheal collapse dogs

**DOI:** 10.3389/fvets.2023.1296087

**Published:** 2023-10-05

**Authors:** Raktham Mektrirat, Thareerat Rueangsri, Waraporn Keeratichandacha, Sasiwimon Soonsawat, Chavalit Boonyapakorn, Wanpitak Pongkan

**Affiliations:** ^1^Department of Veterinary Biosciences and Veterinary Public Health, Faculty of Veterinary Medicine, Chiang Mai University, Chiang Mai, Thailand; ^2^Integrative Research Center for Veterinary Circulatory Sciences, Faculty of Veterinary Medicine, Chiang Mai University, Chiang Mai, Thailand; ^3^Veterinary Diagnostic Laboratory, Faculty of Veterinary Medicine, Chiang Mai University, Chiang Mai, Thailand; ^4^Department of Companion Animal and Wildlife Clinic, Faculty of Veterinary Medicine, Chiang Mai University, Chiang Mai, Thailand; ^5^Veterinary Cardiopulmonary Clinic, Small Animal Hospital, Faculty of Veterinary Medicine, Chiang Mai University, Chiang Mai, Thailand; ^6^Center of Excellence in Veterinary Biosciences, Faculty of Veterinary Medicine, Chiang Mai University, Chiang Mai, Thailand

**Keywords:** polyunsaturated fatty acid EAB-277^®^, heart rate variability, tracheal collapse, inflammatory marker, malondialdehyde, dog

In the published article, there was an error in the Funding statement:

This work was partially supported by VetzPetz Asia Company and Chiang Mai University (CMU) (WP, RM, and CB), which provided the necessary budget through the Center of Excellence in Veterinary Biosciences, Faculty of Veterinary Medicine, Chiang Mai University, R000029941 (WP), and the Faculty of Veterinary Medicine, Chiang Mai University (WP, RM, and CB). The authors declare that this study received funding from VetzPetz Asia Company. The funder was not involved in the study design, collection, analysis, interpretation of data, the writing of this article or the decision to submit it for publication.

The correct Funding statement appears below.

This work was partially supported by Pharmalink International Limited and Chiang Mai University (CMU) (WP, RM, and CB), which provided the necessary budget through the Center of Excellence in Veterinary Biosciences, Faculty of Veterinary Medicine, Chiang Mai University, R000029941 (WP), and the Faculty of Veterinary Medicine, Chiang Mai University (WP, RM, and CB). The authors declare that this study received funding from Pharmalink International Limited. The funder was not involved in the study design, collection, analysis, interpretation of data, the writing of this article or the decision to submit it for publication.

The authors apologize for this error and state that this does not change the scientific conclusions of the article in any way. The original article has been updated.

